# Itaconate induces tolerance of *Staphylococcus aureus* to aminoglycoside antibiotics

**DOI:** 10.3389/fmicb.2024.1450085

**Published:** 2024-09-30

**Authors:** Runping Zhao, Lei Xu, Jieyun Chen, Yanxian Yang, Xilong Guo, Min Dai, Guo-Bao Tian, Li-Na Qin

**Affiliations:** ^1^School of Laboratory Medicine, Chengdu Medical College, Chengdu, China; ^2^Zhongshan School of Medicine, Advanced Medical Technology Center, The First Affiliated Hospital, Sun Yat-sen University, Guangzhou, China; ^3^Department of Immunology, School of Medicine, Sun Yat-Sen University, Shenzhen, China; ^4^State Key Laboratory of Oncology in South China, Sun Yat-sen University Cancer Center, Guangzhou, China; ^5^Department of Pharmacy, The Fifth Affiliated Hospital, Sun Yat-sen University, Zhuhai, China; ^6^Key Laboratory of Tropical Diseases Control, Sun Yat-sen University, Ministry of Education, Guangzhou, China; ^7^Faculty of Forensic Medicine, Zhongshan School of Medicine, Sun Yat-sen University, Guangzhou, China; ^8^Guangdong Province Translational Forensic Medicine Engineering Technology Research Center, Sun Yat-sen University, Guangzhou, China

**Keywords:** *Staphylococcus aureus*, itaconate, antibiotic tolerance, aminoglycosides, immune metabolite

## Abstract

**Introduction:**

*Staphylococcus aureus* is one of the chief pathogens that cause chronic and recurrent infections. Failure of the antibiotics to curb the infections contributes to relapse and is an important reason for the high mortality rate. Treatment failure may also be due to antibiotic tolerance. Accumulating evidence suggests that t the host immune environment plays an important role in inducing antibiotic tolerance of *S. aureus*, but research in this area has been limited.

**Methods:**

In this study,the minimum inhibitory concentration (MIC) of the antibiotics against *S. aureus* was determined using the standard broth microdilution method.The study evaluated whether itaconate induces antibiotic tolerance in *S. aureus* through an antibiotic bactericidal activity assay.The effect of itaconate on the growth of *S. aureus* was evaluated by monitoring the growth of *S. aureus* in medium supplemented with itaconate. Additionally, RNA sequencing and metabolomics analyses were used to determine transcriptional and metabolic changes in *S. aureus* when exposed to itaconate.

**Results and discussion:**

According to the study,we found that the immune metabolite itaconate can induce tolerance in both methicillin-resistant and -susceptible *S. aureus* to aminoglycosides. When *S. aureus* was exposed to itaconate, its growth slowed down and transcriptomic and metabolomic alterations associated with decreased energy metabolism, including the tricarboxylate cycle, glycolysis, pyruvate metabolism, and arginine biosynthesis, were observed. These changes are associated with aminoglycoside tolerance. This study highlights the role of immune signaling metabolites in bacterial antibiotic tolerance and suggests new strategies to improve antibiotic treatment by modulating the host immune response and stimulating the metabolism of bacteria.

## 1 Introduction

The ineffectiveness of antibiotic treatment is an important reason for the high mortality rate and the relapse of bacterial infections. In addition to the development of antibiotic resistance, there is growing evidence that ineffective antibiotic treatment is often attributed to bacterial tolerance and persistence (Labreche et al., 2013; Gimza and Cassat, 2021; Herren et al., 2022). Tolerance is the ability of a bacterial population to survive a transient exposure to high concentrations of antibiotics (Brauner et al., 2016). Tolerant bacteria have increased survival time in the presence of high concentrations of antibiotics without an increase in the minimum inhibitory concentration (MIC) (Balaban et al., 2019). However, persistence is the ability of a subset of a clonal bacterial population to survive exposure to high concentrations of antibiotics (Gefen and Balaban, 2009). The majority of the bacterial population is rapidly killed while a subpopulation persists for a much longer period of time (Balaban et al., 2019). Tolerance and persistence are similar phenomena of increased survival in the presence of an antibiotic, and the two terms are often interchangeable in a study focusing on only a qualitative understanding of the molecular mechanisms (Meylan et al., 2018). Bacteria have increased tolerance when exposed to physiological conditions that lead to reduced growth rates, induction of bacterial dormancy, and decreased metabolism *in vivo* (Brauner et al., 2016). Furthermore, it has been demonstrated that tolerance promotes the development of resistance (Levin-Reisman et al., 2017; Liu et al., 2020; Santi et al., 2021). Therefore, gaining a deeper insight into the factors that induce tolerance *in vivo* can not only improve treatment strategies but also delay the emergence of resistance.

*Staphylococcus aureus* is a prominent human pathogen worldwide, frequently associated with recurrent infections and a high mortality rate (Ikuta et al., 2022). Because *S. aureus* can colonize and proliferate in a variety of host niches, there are many factors that can induce it to develop tolerance. Macrophage-produced reactive oxygen species (ROS) can inhibit the tricarboxylic acid (TCA) cycle of *S. aureus*, leading to the development of antibiotic tolerance (Rowe et al., 2020). In addition, the activation of the NOD-, LRR-, and pyrin domain-containing protein 3 (NLRP3) induces *S. aureus* tolerance to antibiotics via glucose limitation and adenosine triphosphate (ATP) depletion (Beam et al., 2023). This suggested that the host immune environment plays an important role in inducing antibiotic tolerance of *S. aureus*. Therefore, a deeper understanding of how the host immune environment induces antibiotic tolerance is a prerequisite for optimizing therapeutic strategies to combat relapsed *S. aureus* infections.

Recent studies have identified that *S. aureus* infection induces metabolic reprogramming of host myeloid immune cells, resulting in the release of itaconate, an immune metabolite, into the local environment of the infection (Tomlinson et al., 2021; Tomlinson et al., 2023). Itaconate is converted from *cis*-aconitate by the enzyme *cis*-aconitate decarboxylase (ACOD1), encoded by the immunoresponsive gene 1 (*Irg1*), in the mitochondrial matrix of myeloid immune cells (Strelko et al., 2011; Michelucci et al., 2013). Itaconate primarily exerts its anti-inflammatory effect by either activating the anti-inflammatory pathway or inhibiting the inflammatory pathway. For example, itaconate alkylates cysteine residues of Kelch-like ECH-associated protein 1 (KEAP1) to activate the anti-inflammatory transcription factor nuclear factor erythroid 2-related factor 2 (Nrf2) (Mills et al., 2018). Itaconate also alkylates cysteine residues of the NLRP3 inflammasome to abolish its ability to interact with the mitotic kinase NIMA-related kinase 7 (NEK7), a process necessary for inflammasome activation NLRP3 (Hooftman et al., 2020). Similarly, itaconate can also inhibit Janus kinase 1 (JAK1) kinase and TET DNA dioxygenase activity to dampen inflammatory responses (Chen et al., 2022; Runtsch et al., 2022). Beyond its immunomodulatory properties, itaconate has been reported to reprogram *S. aureus* metabolism, leading to biofilm formation and acidic, oxidative, and electrophilic stress responses in *S. aureus* (Tomlinson et al., 2021; Loi et al., 2023; Tomlinson et al., 2023). However, whether itaconate influences the effectiveness of antibiotic treatment remains unclear. We showed that itaconate can induce tolerance in both methicillin-resistant *S. aureus* (MSSA) and methicillin-susceptible *S. aureus* (MRSA) to aminoglycoside antibiotics, suggesting new strategies to improve antibiotic treatment for *S. aureus* infections by modulating the host immune response and stimulating the metabolism of bacteria.

## 2 Materials and methods

### 2.1 Bacterial culture and growth conditions

*Staphylococcus aureus* ATCC29213 was purchased from ATCC (Manassas, VA, USA) and *S. aureus* BA011, SAU29, and SAU49 were isolated from the patients with lower respiratory tract infections (BA011 is MRSA, SAU29, and SAU49 are MSSA). *S. aureus* was cultured overnight on mannitol salt agar for enumeration. Mueller Hinton broth (MHB) or trypticase soy broth (TSB) were used for bacterial liquid culture. All clinical strains were isolated from another study, which was registered on the Chinese Clinical Trial Registry (Registration number ChiCTR1900023317, 5/22/2019) and approved by the Research Ethics Committee of the hospital (approval number K51-2, 2018).

### 2.2 Growth kinetics

The *S. aureus* strains were grown in MHB or TSB medium overnight. The cultures were pelleted, washed once with phosphate-buffered saline (PBS), adjusted to an OD600 of 0.5 with PBS, and diluted 1:10. Then, 10 μL of the diluted culture was added to 90 μL of either TSB or TSB supplemented with 0–10 mM itaconate. The pH of the medium was adjusted to 7.2 with 10 M sodium hydroxide. The OD600 of the culture within 16 h was determined using a microplate reader (BioTek). Three replicates were analyzed for each strain.

### 2.3 Antimicrobial susceptibility testing

The MIC of the antibiotics against *S. aureus* was determined using the standard broth microdilution method, according to the CLSI 2024 guidelines. In brief, all antibiotics were two-fold serially diluted in cation-adjusted Mueller Hinton broth (CAMHB) and 90 μL of this solution was mixed with 10 μL of diluted culture containing approximately 1.5 × 10^6^ CFU/mL in a 96-well microtiter plate. After 16 h of incubation at 37°C, the MIC values were defined as the lowest concentrations of antibiotics with no visible bacterial growth by the OD600 measured.

### 2.4 Antibiotic killing assays

The *S. aureus* strains were grown in MHB overnight. The cultures were then diluted 1:1000 in MHB with or without 10 mM itaconate and refreshed for 5 h. Bacterial cells were harvested, washed once with sterile PBS via centrifugation (5,000 × *g* for 5 min), and adjusted with sterile PBS to ~5 × 10^7^ CFU/mL. Vancomycin, ciprofloxacin, gentamicin, amikacin, or kanamycin were added to *S. aureus* at a concentration of 10 μg /mL. Cultures were incubated at 37°C with shaking (220 rpm). Every 2 h, aliquots were washed with PBS once and serially diluted 10-fold in PBS to enumerate the survivors by colony-forming units (CFU) count.

### 2.5 THP-1 cell stimulate and siRNA transfection

THP-1 cells were cultured at 37°C and 5% CO_2_ in RPMI 1640 supplemented with l-glutamine, 10% heat-inactivated fetal bovine serum, and 1% penicillin/streptomycin. Two days before transfection, cells were reseeded at 1 × 10^6^ cells/well in cell culture dishes in a medium supplemented with 1 μM phorbol myristate acetate (PMA). After 1 day, the media was replaced with 500 μL RPMI 1640 without penicillin/streptomycin. siRNA-targeting and non-targeting controls (100 nM) prepared by RiboBio (Guangzhou, China) were used to transfect into cells. Lipofectamine 3,000 (Thermo Fisher Scientific, Waltham, MA, USA) was used to increase the transfection efficiency according to the manufacturer’s protocol.

### 2.6 THP-1 cell infection and bacterial plate-killing assays

After 12 h of siRNA transfection, the medium in the wells reserved for infection was replaced with RPMI 1640 containing l-glutamine and 10% heat-inactivated fetal bovine serum. For infection, the cells were incubated for 7 h at 37°C at a multiplicity of infection (MOI) of “1” using *S. aureus* strains suspended in PBS. After infection, macrophages were collected and RNA was extracted for RT-qPCR analysis. Extracellular bacterial cells were collected from the medium and washed once with sterile PBS by centrifugation (5,000 × *g* for 5 min). Bacterial cells were treated with 20 μg/mL of gentamicin for 2 h. Aliquots were serially diluted 10-fold in PBS and plated to determine bacterial viability by the CFU count. Percent survival was calculated based on the ratio of the number of bacteria surviving after antibiotic treatment to the number of bacteria surviving without any treatment.

### 2.7 RT-qPCR detection of *Irg1* expression of THP-1

FastPure^®^ Cell/Tissue Total RNA Isolation Kit V2 (Vazyme, Nanjing, China) was used to extract total RNA according to the manufacturer’s protocol. The total RNA was reverse transcribed into cDNA and the cDNA levels of *Irg1* were quantified according to a previously published protocol (Feng et al., 2022). The signals were normalized to fold change of the housekeeping *GAPDH* transcript and analyzed according to the ΔΔCt method. The primers used for RT-qPCR are listed in Supplementary Table 3.

### 2.8 RNA-seq analysis

A total of six samples were used for RNA-seq analysis (three biological replicates were performed for each group). The *S. aureus* ATCC2213 strain was grown in MHB with or without 10 mM itaconate until the late exponential phase (5 h). Total RNA was extracted using the RNAprep Pure Cell/Bacteria Kit (TIANGEN Biotech, Beijing, China) according to the manufacturer’s protocol. The library prepared and sequenced on an Illumina Novaseq platform (Nonogene, Guangzhou, China). The reference genome and gene model annotation files were downloaded directly from the National Centre for Biotechnology Information (NCBI) website. Fastqc (v0.11.8) was used to remove the low-quality paired-end reads. Then, we used HISAT2 (v2.1.0) to create an index file for the reference genome (*S. aureus* ATCC2213). The processed reads were mapped to the reference genome using HISAT2(v2.1.0). FeatureCounts (v1.6.4) were used to calculate gene expression and the edgeR package DESeq2(v1.38.3) was used to identify differentially expressed genes (DEGs) between the itaconate-adapted and control groups. We corrected all the statistical test results for multiple testing with the Benjamini–Hochberg false discovery rate (FDR ≤ 0.05) and determined the significant differences in gene expression with FDR <0.05 and absolute Log2FC (|Log2FC| > 1.0). ClusterProfiler (v4.6.2) and pheatmap (v1.0.12) were used to perform Kyoto Encyclopedia of Genes and Genomes (KEGG) and cluster analyses, respectively. We created a volcano plot of the DEGs and a bubble diagram of the KEGG enrichment analysis results using the edgeR package ggplot2 (v3.4.4).

### 2.9 Metabolomics analysis

A total of 12 samples were used for metabolomics analysis (six biological replicates were performed for each group). The *S. aureus* ATCC2213 was grown in MHB with or without 10 mM itaconate until the late exponential phase (5 h). The intracellular metabolites were extracted according to a previously published protocol (Zhao et al., 2024). Then, a 80-μL sample of the supernatant was transferred for liquid chromatography-mass spectrometry (LC–MS) analysis. The original data file acquired from LC–MS was converted into the mzML format by ProteoWizard software. For two-group analysis, we corrected all statistical test results for multiple testing with the Benjamini–Hochberg false discovery rate (FDR ≤ 0.05) and determined differential metabolites with FDR <0.05 and absolute Log2FC (|Log2FC| ≥ 1.0 and |Log2FC| ≤ 0.5). Identified metabolites were annotated using the KEGG compound database and annotated metabolites were mapped to the KEGG pathway database. Significantly enriched pathways were identified based on the *p*-value of a hypergeometric test, which was considered statistically significant when the *p* < 0.05.

### 2.10 ATP detection

The *S. aureus* ATCC2213 was grown in MHB with or without 10 mM itaconate until the late exponential phase (5 h). The Enhanced ATP Assay Kit (Beyotime, Shanghai, China) was used to detect intracellular ATP according to the manufacturer’s protocol. In brief, bacterial cells were harvested, washed once with sterile PBS via centrifugation (5,000 × *g* for 5 min), and adjusted with sterile PBS to 2 × 10^7^ CFU/mL. After 400 μL of the bacterial cells were pelleted, 200 μL of Buffer C (provided in the kit) was added to lyse the cells. After lysis, the solution was centrifuged at 4°C 12,000× g for 5 min. The supernatant was used for subsequent determination. A 100 μL of ATP detection working solution was added to the 96-well microtiter plate placed at room temperature for 3–5 min. Then, 20 μL of the sample or standard was added into the 96-well microtiter plate and quickly mixed with a micropipette. The relative light unit (RLU) of luminescence was measured with a microplate reader (Biotek synergy2).

### 2.11 Texas red- gentamicin uptake

The ATCC2213 was grown in MHB overnight. The cultures were then diluted 1:1000 in MHB with or without 10 mM itaconate and refreshed for 5 h. Then, bacterial cells were treated with the Texas Red–gentamicin at a final concentration of 10 μg/mL. After 1 h, an aliquot of cells was removed, washed twice in PBS, and 1 μL of the sample was covered with a 1% agarose pad. The samples were subjected to fluorescence microscopy for detecting signals at 555 nm excitation (555 nmex). The fluorescence of tetramethylrhodamine (TRTC) at 555 nmex was recorded by Olympus BX63 fluorescence microscopy on a phase-contrast microscope equipped with a 100 × oil immersion objective and a xenon lamp. ImageJ software was used to calculate the average fluorescence intensity of the collected images.

### 2.12 Statistical analysis

Statistical analysis was performed using GraphPad Prism 9.4.0 for Windows. All data are presented as mean ± SD. Unpaired two-tailed student’s *t*-tests and non-parametric one-way ANOVA were used to calculate the value of *p <* 0.05 was considered statistically significant.

## 3 Results

### 3.1 Itaconate induces aminoglycoside antibiotic tolerance in *Staphylococcus aureus* ATCC29213

**Figure 1:**
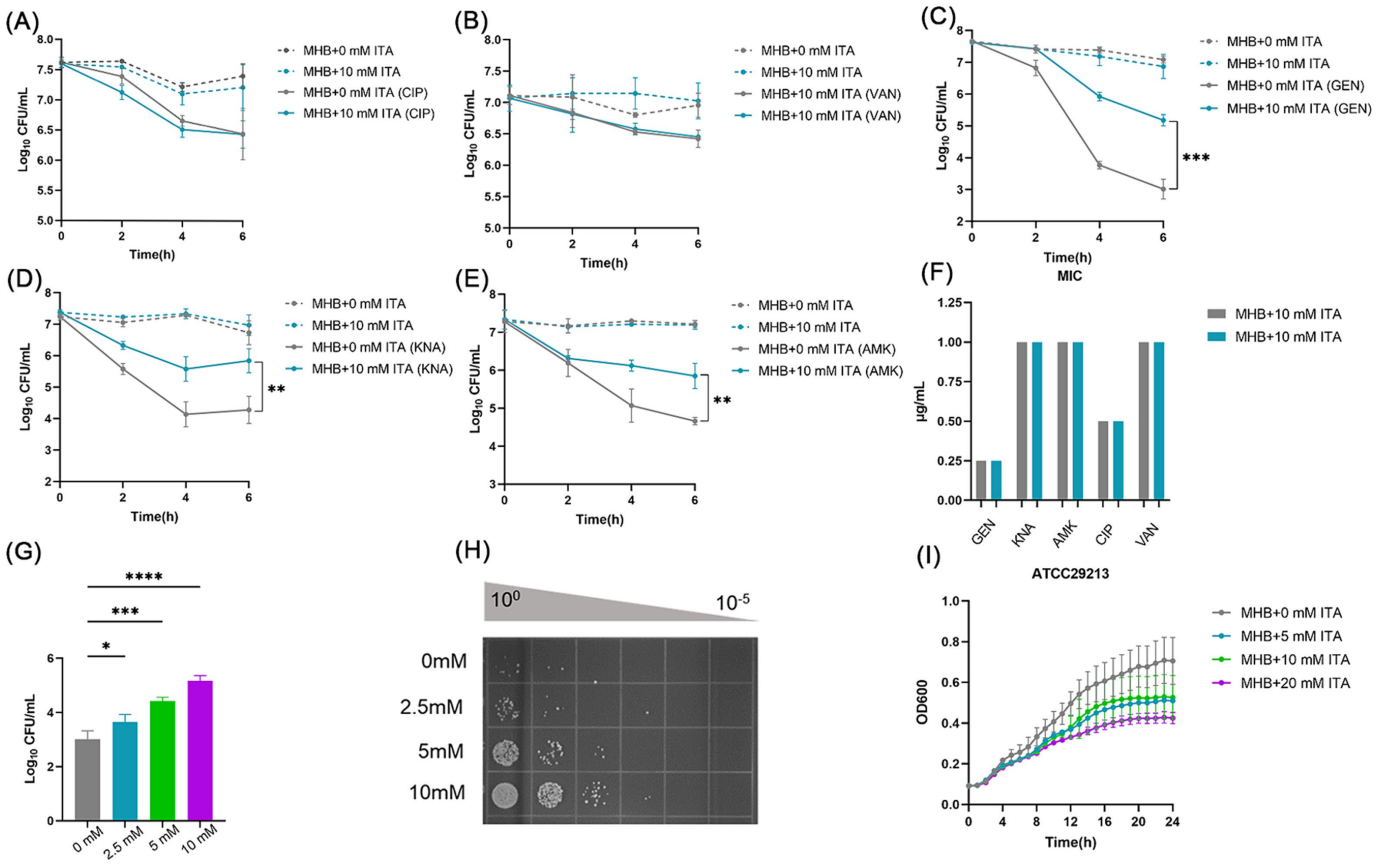
Itaconate induces aminoglycoside antibiotic tolerance in *S. aureus.* **(A–E)** The time-dependent killing pattern of ATCC29213 in the MHB-grown group and the itaconate-adapted group over 6 h. The graphs show log_10_ CFU /ml of *S. aureus* challenged with 10 μg/mL antibiotic [ciprofloxacin (CIP), vancomycin (VAN), gentamycin (GEN), kanamycin (KNA), and amikacin (AMK)]. **(F)** MIC of antibiotics against ATCC29213 in the MHB-grown group and the itaconate-adapted group **(G–H)** Log_10_ CFU /ml of ATCC29213 (grown to the late exponential phase in MHB medium with 0–10 mM itaconate) challenged with 10 μg/mL gentamycin. **(I)** Representative growth curves of ATCC29213 in MHB supplemented with itaconate (0–20 mM). Data represent the mean ± SD. Statistical significance was determined by unpaired *t*-test (two-tailed) or one-way ANOVA with Sidak’s multiple comparison. ^*^p < 0.05, ^**^p < 0.01, ^***^p < 0.001, ^****^p < 0.0001.

To investigate the impact of itaconate on the efficacy of antibiotic treatment for *S. aureus*, we firstly performed antibiotic bactericidal activity assays of *S. aureus* standard strain (ATCC29213) cultured in MHB (“MHB-grown”) or MHB supplemented with 10 mM itaconate (“itaconate-adapted”), a concentration considered to represent exposure levels of *S. aureus* within the host (Michelucci et al., 2013; Tomlinson et al., 2021). The time-dependent killing pattern over 6 h and the survival rate were observed (Figures 1A–C and Supplementary Figure S1A). Although there was no significant difference following ciprofloxacin and vancomycin treatment, the levels of bacterial survival following 6 h of gentamicin treatment significantly increased by approximately two orders of magnitude in itaconate-adapted *S. aureus*. Because gentamycin is an aminoglycoside, other aminoglycosides, including amikacin and kanamycin, were also tested with bactericidal activity assays. Higher levels of bacterial survival were observed in itaconate-adapted *S. aureus* (Figures 1D,E and Supplementary Figure S1A), as observed with gentamycin. We also performed antimicrobial susceptibility test to determine the MIC of antimicrobial agents against the *S. aureus* cultured in MHB or MHB supplemented with 10 mM itaconate. Antimicrobial susceptibility test results showed that the MIC was not altered by itaconate (Figure 1F), suggesting that itaconate increased the tolerance but not the resistance of *S. aureus* to aminoglycosides. So a longer exposure to an antibiotic is needed to produce bacterial death in a tolerant strain as produced in a susceptible strain (Brauner et al., 2016). So, we calculated the survival rate after being challenged with antibiotics for 24 h. There was no difference between the itaconate-adapted group and the MHB-grown group (Supplementary Figure S1B). We examined whether itaconate-induced tolerance to aminoglycosides in *S. aureus* was concentration-dependent. The ATCC29213 was grown in MHB containing 0–10 mM itaconate, and the survivors were enumerated following the gentamicin challenge for 6 h. We found that the survivors increased with the concentration of itaconate (Figures 1G,H). Previous studies have shown that conditions that decrease the rate of growth can increase tolerance to antibiotics. Therefore, we monitored the growth of ATCC29213 in MHB and TSB supplemented with itaconate at concentrations ranging from 0 to 20 mM. As shown in (Figure 1I and Supplementary Figure S1C), with the elevation of itaconate concentration, the proliferation rate of *S. aureus* gradually attenuated. Taken together, these results suggested that itaconate may induce tolerance to aminoglycosides by slowing the growth of *S. aureus* in a concentration-dependent manner.

### 3.2 Itaconate induces aminoglycoside antibiotic tolerance in clinical MSSA and MRSA isolates

**Figure 2:**
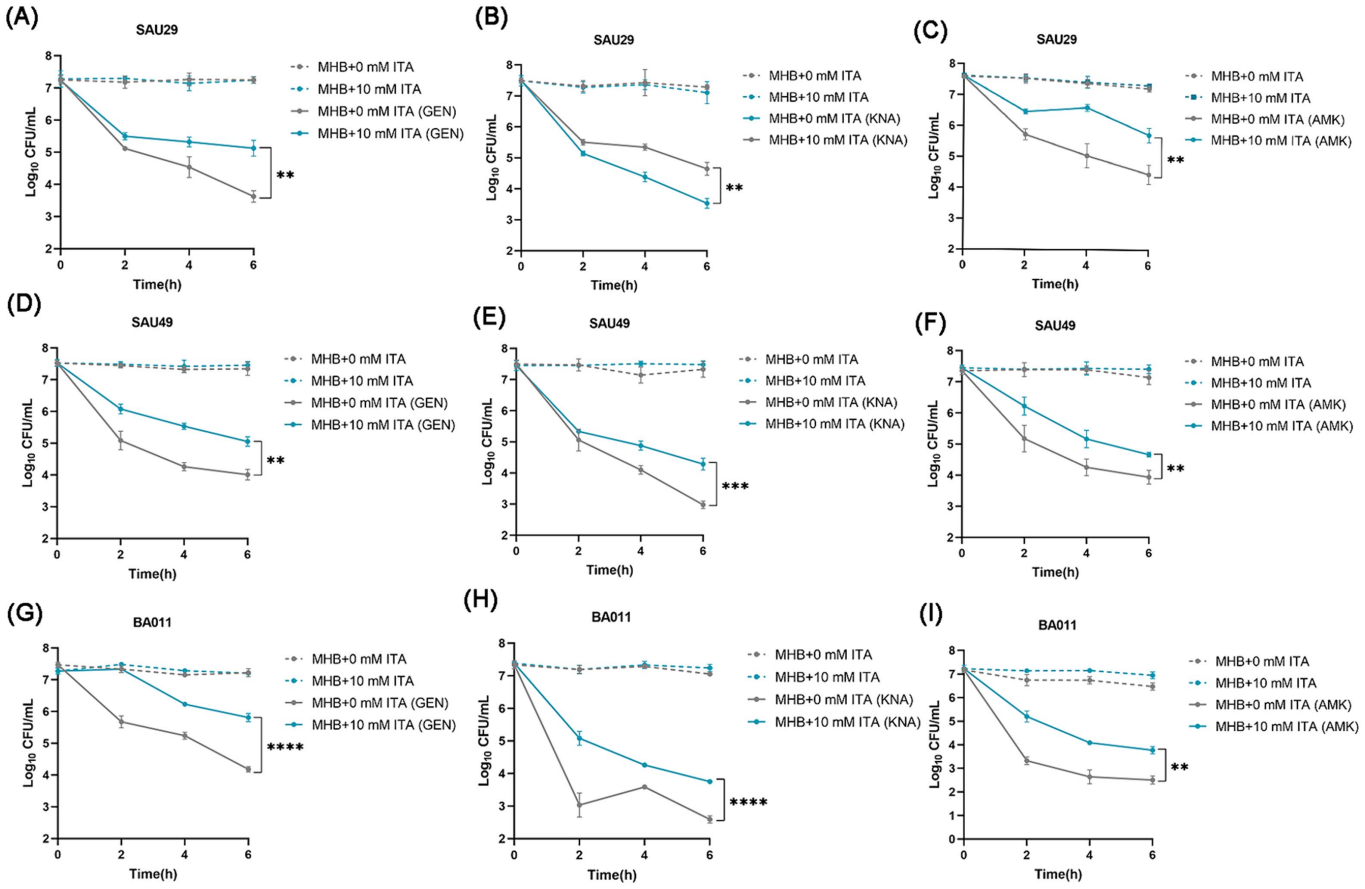
Itaconate induces aminoglycoside antibiotic tolerance in methicillin-resistant and -susceptible clinical *S. aureus* isolates. **(A–I)** The time-dependent killing pattern of SAU29, SAU49, and BA011 in the MHB-grown group and the itaconate-adapted group over 6 h. The graphs show log_10_ CFU /ml of *S. aureus* challenged with 10 μg/mL antibiotic [gentamycin (GEN), kanamycin (KNA), and amikacin (AMK)]. Data represent mean ± SD. Statistical significance was determined by unpaired *t*-test (two-tailed).

To confirm its clinical correspondence that itaconate induces tolerance of *S. aureus* to aminoglycosides, we investigated the effect of itaconate on clinical *S. aureus* isolates SAU29 and SAU49. SAU29 and SAU49 belong to different ST types, ST5 and ST59. ST5 was one of the dominant hospital-associated genotypes worldwide, and ST59 is the predominant community-associated clone in Asia (Xu and Yuan, 2022). Through antibiotic bactericidal activity assays, we observed that there was no difference in survival between the MHB-grown group and the itaconate-adapted group after 6 h of ciprofloxacin and vancomycin treatment (Supplementary Figures S2A–D). However, after 6 h of aminoglycoside antibiotic treatment, the survival in itaconate-adapted group was significantly more than that in the MHB-grown group (Figures 2A–F). MRSA has spread worldwide and poses a serious threat to public health systems. We also performed antibiotic bactericidal activity assays of a clinical MRSA isolate (BA011). Similar to clinical MSSA isolates, there was no difference in survival between the MHB-grown group and the itaconate-adapted group after 6 h of ciprofloxacin and vancomycin treatment (Supplementary Figures S2E,F), but the survival in itaconate-adapted group was significantly more than that in MHB grown after 6 h of aminoglycoside antibiotic treatment (Figures 2G–I). In addition, we determined the MIC of antibiotics against these clinical MSSA and MRSA isolates. As Table 1 shows, the MIC of antibiotics against the clinical isolates was not influenced by itaconate treatment. Moreover, we also determined the growth curve of these clinical MSSA and MRSA isolates in TSB and MHB supplemented with itaconate in a concentration range of 0–20 mM. Their proliferation rate gradually attenuated with the elevation of itaconate concentration (Supplementary Figures S2G–L). These results suggested that itaconate can induce tolerance to aminoglycosides in *S. aureus*. These results indicate that itaconate induces aminoglycoside antibiotic tolerance in clinical MSSA and MRSA isolates.

**Table 1:** Minimum inhibitory concentrations of antibiotics tested.

Antibiotic	MIC (μg/mL) Oxacillin	Ciprofloxacin	Vancomycin	Gentamycin	Amikacin	Kanamycin
ATCC29213 (MHB)	0.25	0.5	1	0.25	1	1
ATCC29213 (MHB + 10 mM ITA)	0.25	0.5	1	0.25	1	1
SAU29 (MHB)	0.25	0.25	1	0.5	2	1
SAU29 (MHB + 10 mM ITA)	0.25	0.25	1	0.5	2	1
SAU49 (MHB)	0.25	0.25	1	0.5	2	1
SAU49 (MHB + 10 mM ITA)	0.25	0.25	1	0.5	2	1
BA011 (MHB)	8	0.5	1	0.25	2	2
BA011 (MHB + 10 mM ITA)	8	0.5	1	0.25	2	2

### 3.3 Macrophage-produced itaconate induces tolerance to aminoglycosides in *Staphylococcus aureus*

**Figure 3:**
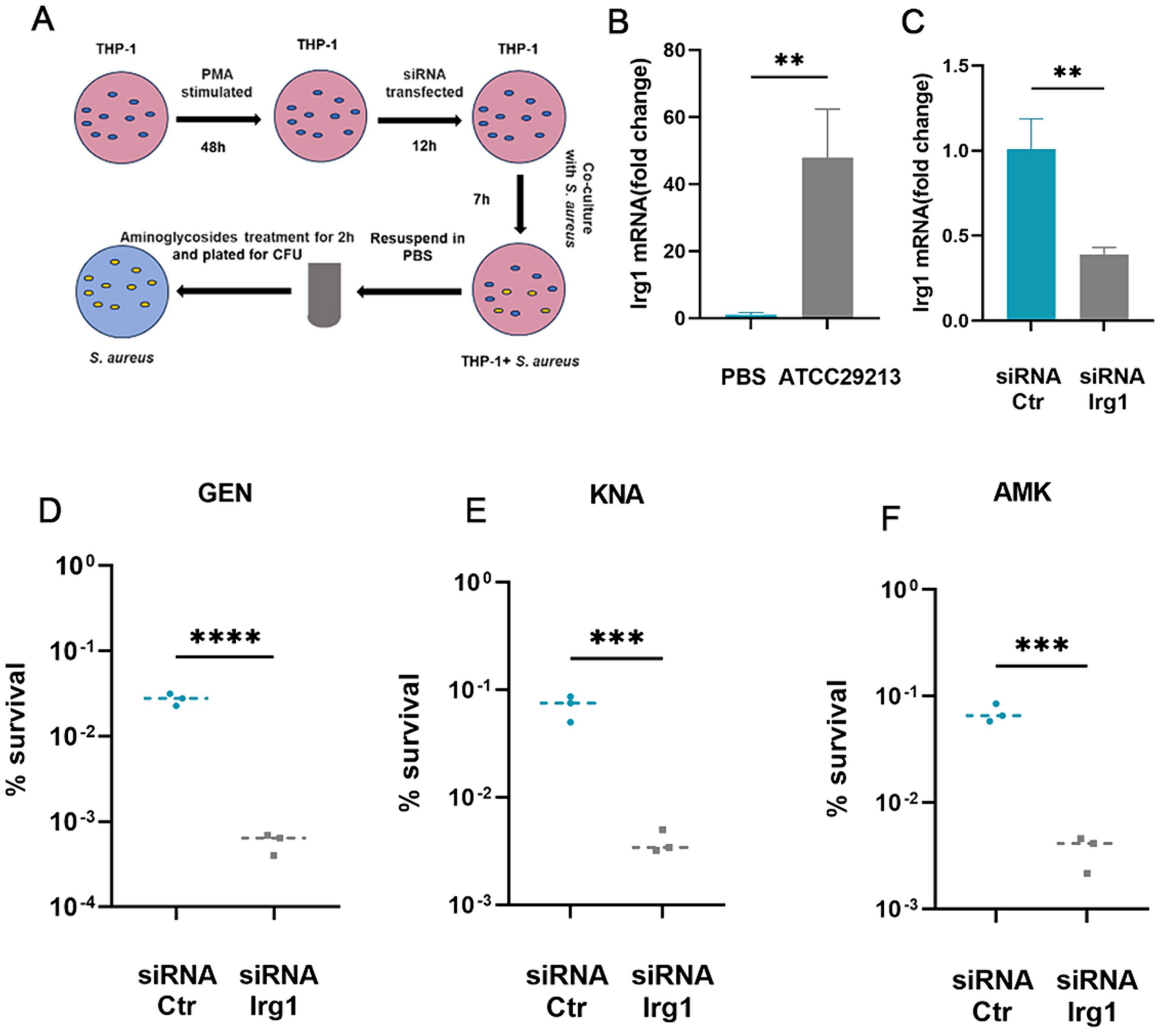
Macrophage-produced itaconate induces tolerance to aminoglycosides in *S. aureus*. **(A)** A schematic illustrating the investigative protocol for examining whether macrophage-produced itaconate can induce aminoglycoside tolerance in *S. aureus*. **(B)** Levels of *Irg1* mRNA in resting (PBS) or *S. aureus*-infected THP-1 macrophages. **(C)** Levels of *Irg1* mRNA in *S. aureus*-infected THP-1 macrophages transfected with either siRNA specific for *Irg1* (siRNA *Irg1*) or with siRNA control (siRNA Ctr). **(D–F)** *S. aureus* was co-cultivated with THP-1 macrophages transfected with either siRNA specific for irg1 or with siRNA Ctr followed by treatment with 20 ug/mL gentamycin (GEN), amikacin (AMK), and kanamycin (KNA) for 2 h. Percent survival was calculated based on the ratio of the number of bacteria surviving after antibiotic treatment to the number of bacteria surviving without any treatment. Data represent mean ± SD. Statistical significance was determined by unpaired t-test (two-tailed). ^*^p < 0.05, ^**^p < 0.01, ^***^p < 0.001, ^****^p < 0.0001.

Itaconate is mainly produced by myeloid immune cells during *S. aureus* infection (Tomlinson et al., 2021; Tomlinson et al., 2023). Therefore, we examined the effects of macrophage-derived itaconate on aminoglycoside tolerance in *S. aureus* (Figure 3A). First, we performed RT-qPCR to analyze the mRNA expression of *Irg1*, which encodes the itaconate-producing *cis*-aconitate decarboxylase in THP-1 cells. Infection with *S. aureus* resulted in a ~ 48-fold increase in the mRNA expression level in *Irg1* (Figure 3B). Next, we designed an siRNA that specifically targeted the coding sequence in the Irg1 mRNA to inhibit its expression and achieved an approximately 60% decrease in *Irg1* mRNA levels compared to the non-specific siRNA control (Figure 3C). Next, *S. aureus* was co-cultured with macrophages that had been transfected overnight with siRNA Irg1 or non-specific siRNA control for 7 h. Antibiotic bactericidal activity assays were performed to assess the effect of macrophage-derived itaconate on aminoglycoside tolerance of extracellular *S. aureus*. The results showed that fewer *S. aureus* cells survived in the siRNA *Irg1* group after 2 h of challenge with gentamycin, amikacin, or kanamycin (Figures 3D–F).

### 3.4 *Staphylococcus aureus* exposed to itaconate exhibit an altered transcriptomic profile

**Figure 4:**
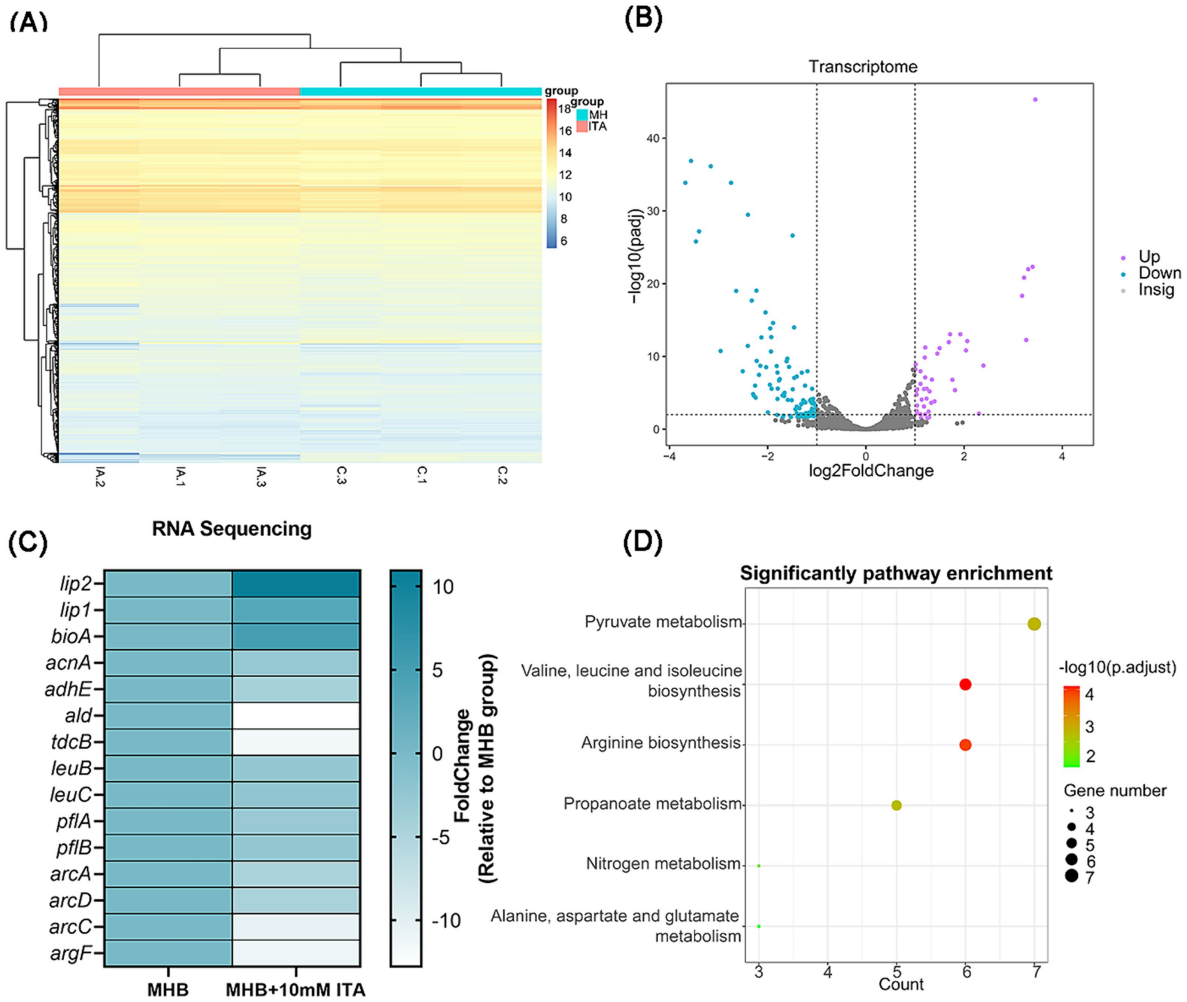
*S. aureus* exposed to itaconate exhibit an altered transcriptomic profile. **(A)** Heat map of DEGs of *S. aureus* between the MHB-grown group and the itaconate-adapted group. **(B)** Volcano map of DEGs of *S. aureus* between the MHB-grown group and the itaconate-adapted group. Significantly upregulated and downregulated genes are shown in purple and blue, respectively. **(C)** The significantly upregulated and downregulated genes in *S. aureus* exposed to itaconate. **(D)** KEGG pathways of downregulated DEGs of *S. aureus* in the itaconate-adapted group.

To understand how itaconate induces aminoglycoside tolerance in *S. aureus*, we performed transcriptome analysis of MHB-grown and itaconate-adapted *S. aureus* ATCC29213. There were global changes in the *S. aureus* transcriptome upon exposure to itaconate (Figure 4A); 45 differentially expressed genes (DEGs) were upregulated and 99 DEGs were downregulated in the itaconate-adapted group (Figure 4B). Among these (Figure 4C), triacylglycerol lipase-related genes *lip1* (3.39-fold) and *lip2* (10.93-fold) were significantly upregulated. However, the expression of the *ald* (12.79-fold) encoding l-alanine dehydrogenase, the *tdcB* (11.82-fold) encoding threonine dehydrase, and the *leuC* (2.66-fold) and *leuB* (2.30-fold) encoding the large and small subunits of isopropyl malate isomerase in the leucine biosynthesis pathway was significantly downregulated. The expressions of *pflA* (3.04-fold) and *pflB* (2.65-fold), which are involved in pyruvate metabolism, were also significantly downregulated. We also observed that the expression of multiple genes involved in arginine biosynthesis, including *argF* (11.02-fold), *arcA* (4.78-fold), *arcC* (3.47-fold), and *arcD* (4.67-fold), were significantly downregulated. In addition, itaconate decreased the expression of aconitase coding gene *acnA* (2.85-fold) and alcohol dehydrogenase E coding gene *adhE* (4.37-fold), which are involved in the tricarboxylic acid (TCA) cycle and glycolysis, respectively. KEGG analysis revealed that the downregulated genes were predominantly involved in pyruvate metabolism, valine, leucine, isoleucine biosynthesis, and arginine biosynthesis (Figure 4D).

### 3.5 Itaconate decreased the energy metabolism and arginine biosynthesis

**Figure 5:**
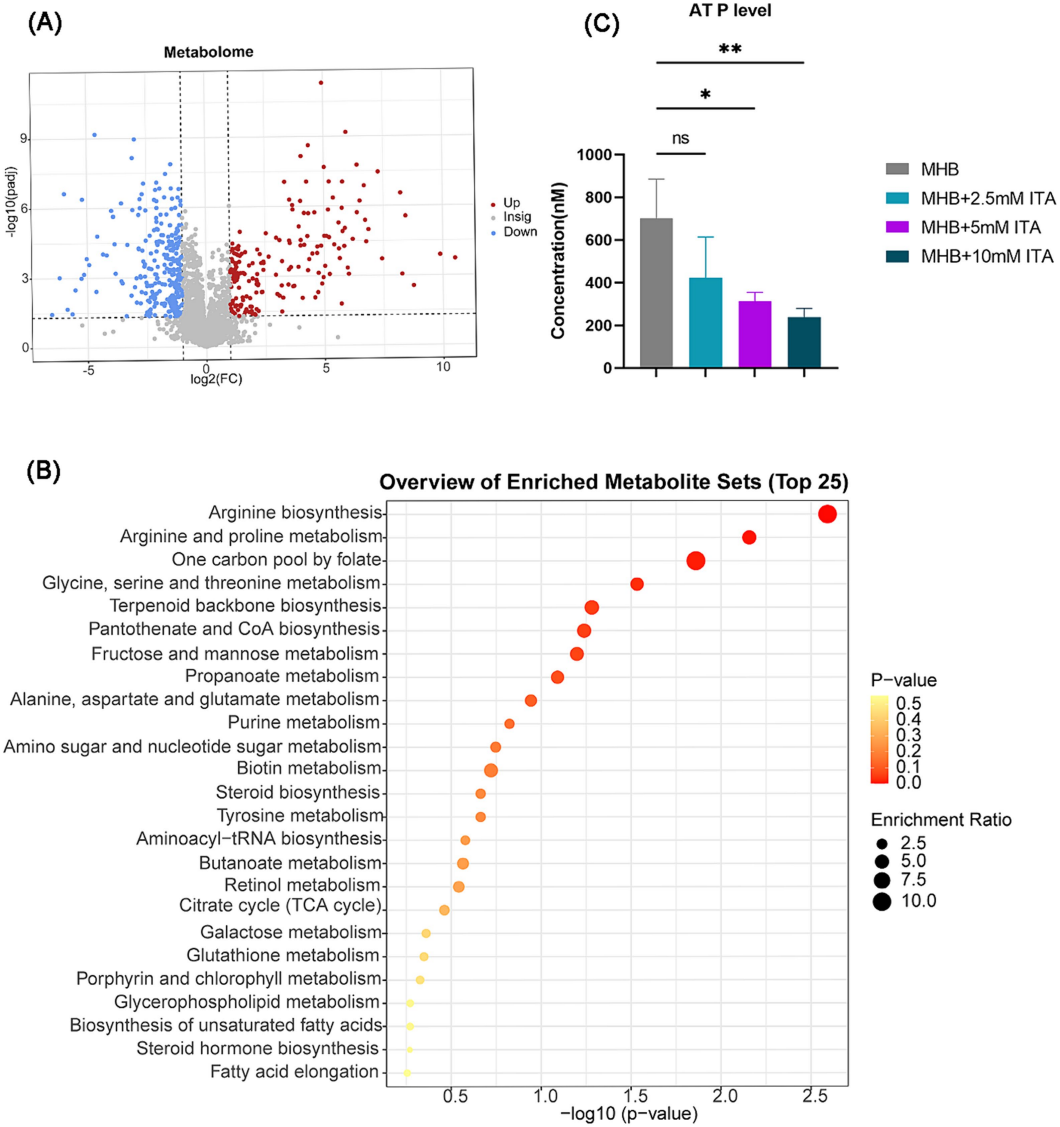
Itaconate decreased the energy metabolism and arginine biosynthesis. **(A)** Volcano maps of DEMs of *S. aureus* between the MHB-grown group and the itaconate-adapted group. Significantly upregulated and downregulated genes are shown in red and blue, respectively. **(B)** KEGG pathways of DEMs between the MHB-grown group and the itaconate-adapted group. **(C)** Intracellular ATP level of *S. aureus* ATCC29213 grown to late exponential phase (5 h) in MH medium with itaconate (0–10 mM). Data represent mean ± SD. Statistical significance was determined by one-way ANOVA with Sidak’s multiple comparison. ^*^p < 0.05, ^**^p < 0.01.

Given that itaconate induced global changes in *S. aureus* in the transcriptome, we also investigated the metabolic changes of ATCC29213 upon exposure to itaconate. A total of 457 differentially expressed metabolites (DEMs) were detected between the MHB-grown group and the itaconate-adapted group, of which 185 were upregulated and 272 were downregulated (Figure 5A). The top 15 upregulated and downregulated metabolites are listed in Supplementary Table S1. KEGG analysis showed that the top 25 pathways also included arginine biosynthesis and the TCA cycle (Figure 5B), which are consistent with the results of transcriptomics analysis. Based on the results of the growth curve test, itaconate has been shown to slow the growth of *S. aureus*. Furthermore, transcriptome and metabolome analyses all indicate that itaconate primarily attenuates the energy metabolism of *S. aureus*. Therefore, we evaluated the intracellular ATP levels in ATCC29213 grown in MHB or MHB supplemented with itaconate at concentrations ranging from 0 to 10 mM. We found that the level of ATP in *S. aureus* decreased with the increase of itaconate concentration (Figure 5C). Given that reduced aminoglycosides uptake by bacteria can diminish the bactericidal effect of aminoglycosides, we also tested the uptake of Texas Red–gentamicin by *S. aureus* in both the itaconate-adapted group and the MHB-grown group. But the mean fluorescence intensity was only slightly reduced in the itaconate-adapted group (Supplementary Figure S3), which indicated that there was no significant difference in the uptake of Texas Red–gentamicin between the itaconate-adapted group and the MHB-grown group. In conclusion, itaconate induces aminoglycosides tolerance in *S. aureus*, potentially through the reduction of energy metabolism (including the TCA cycle, glycolysis, pyruvate metabolism, and arginine biosynthesis), and this process does not depend on reducing the aminoglycoside uptake.

## 4 Discussion

There is a growing recognition that antibiotic tolerance plays a crucial role in the high incidence of bacterial infections and contributes to numerous clinical antibiotic treatment failures (Meylan et al., 2018). In this study, we discovered that the immune metabolite itaconate can induce tolerance in both MSSA and MRSA to aminoglycosides. Our discovery provided a deeper understanding of how the host immune environment drives the formation of antibiotic-tolerant *S. aureus*, which highlights the role of immune metabolites in bacterial antibiotic tolerance.

In this research, we found that itaconate slowed down the growth of *S. aureus*. In reality, it is widely acknowledged that the concept of tolerance, as outlined in literature, is often associated with slow growth (Balaban et al., 2004; Gefen et al., 2008; Amato et al., 2013; Huemer et al., 2021). Tolerance is increased when bacterial growth is impaired, such as due to poor growth conditions, cell location within the intracellular or exposure to reactive oxygen species (ROS) (Conlon et al., 2016; Peyrusson et al., 2020; Peyrusson et al., 2022).To gain insights into the specific molecular mechanisms of itaconate-inducing tolerance of *S. aureus* to aminoglycoside antibiotics, we next profiled the *S. aureus* transcriptome and metabolome. Itaconate has been reported to be a potent inhibitor of isocitrate lyase, downregulate glycolysis, and reduce the expression of aconitase (*acnA*), which catalyzes the conversion of citrate into isocitrate in the second step of the TCA cycle (Sasikaran et al., 2014; Tomlinson et al., 2021). Consistent with this, our results showed that itaconate consistently decreased the expression of *adhE* and *acnA*. Moreover, the differential metabolites between the MHB-grown group and the itaconate-adapted group were also enriched in the TCA cycle through KEGG enrichment analysis. Previous research revealed that disrupting the TCA cycle boosts antibiotic tolerance in *S. aureus* (Huemer et al., 2021). In addition, the downregulated genes were also enriched in pyruvate metabolism. Active pyruvate cycle could increase aminoglycoside efficacy by regulating the TCA cycle to provide respiratory energy (Su et al., 2018). We also observed that the intracellular ATP level of *S. aureus* in the itaconate-adapted was approximately two to three times lower compared to the MHB-grown group. Consistent with this, there was no difference in the uptake of Texas Red–gentamicin by *S. aureus* between the itaconate-adapted group and the MHB-grown group. These indicated that itaconate primarily disrupts the energy metabolic pathway of *S. aureus* rather than complete inhibition. In conclusion, itaconate induces aminoglycoside tolerance in *S. aureus* by slowing down the growth, potentially by disrupting the energy metabolic pathway including the TCA cycle, glycolysis, pyruvate metabolism, and arginine biosynthesis.

The study indicates that itaconate can induce tolerance in *S. aureus*. Itaconate, a metabolite of myeloid immune cells, was initially found to exert an anti-inflammatory effect by modulating inflammatory pathways. However, recent studies have revealed its significant role in host–bacterial interactions. Itaconate can stimulate lysosomal biogenesis by activating the transcription factor EB (TFEB), enhancing the clearance of intracellular bacteria (Zhang et al., 2022). Moreover, itaconate acts as an agonist for oxoglutarate receptor 1 (OXGR1), leading to the induction of Ca^2+^ mobilization, ERK phosphorylation, and endocytosis of the receptor (Zeng et al., 2023). This process enhances respiratory defense against *Pseudomonas aeruginosa.* In addition, itaconate can promote biofilm formation by increasing the production of extracellular polysaccharide (EPS) from *P. aeruginosa* and *S. aureus* (Riquelme et al., 2020; Tomlinson et al., 2021). However, biofilms facilitated the bacterial evasion of host immunity and antibiotic treatment. Combined, these studies suggest that itaconate plays two distinct roles in bacterial infections. Recent research revealed that itaconate is transported from the cytosol to the extracellular space by the ATP-binding cassette transporter G2 (ABCG2) (Chen et al., 2024). Therefore, in the future, screening ABG2 inhibitors to limit the transport from the cytosol to the extracellular space may be an effective method to reduce aminoglycoside tolerance of extracellular *S. aureus* and enhance macrophage clearance of intracellular *S. aureus*. Furthermore, this study demonstrated that itaconate disrupted the energy metabolism of *S. aureus*, which plays an important role in tolerance. Therefore, the specific metabolic stimuli enable the killing of tolerant *S. aureus* with aminoglycosides (Allison et al., 2011).

In summary, our study revealed that the immune metabolite itaconate can induce tolerance in both MSSA and MRSA to aminoglycoside antibiotics. This finding suggests that it may be possible to enhance the efficacy of aminoglycosides against tolerant *S. aureus* by modulating the host’s immune response and specific metabolic stimuli. Nevertheless, our study has several limitations. This study found that itaconate induces aminoglycosides tolerance in *S. aureus* by slowing down the growth, potentially by disrupting the energy metabolic pathway including the TCA cycle, glycolysis, pyruvate metabolism, and arginine biosynthesis. However, how itaconate specifically impacts these metabolic pathways requires further experimental investigations.

## Data Availability

The original contributions presented in the study are included in the article/Supplementary material, further inquiries can be directed to the corresponding authors.
