# Supplementary material for: Itaconate induces tolerance of Staphylococcus aureus to aminoglycoside antibiotics
Source: Front Microbiol. 2024 Sep 30;15:1450085. doi: 10.3389/fmicb.2024.1450085 (PMC11471559; doi:10.3389/fmicb.2024.1450085)
Supplement: Supplementary file 1 [file Data_Sheet_1.docx]

**Table.S1** Differentially expressed genes of MH-grown and itaconate-adapted *S. aureus* ATCC29213.

| GID | name | log2(FC) | *p*.ajusted |
| --- | --- | --- | --- |
| UOO13423.1 |  | -3.67667 | 1.41E^-34^ |
| UOO13422.1 | *tdcB* | -3.56279 | 1.38E^-37^ |
| UOO15646.1 | *argF* | -3.46151 | 1.61E^-26^ |
| UOO15647.1 | *arcC* | -3.39769 | 6.63E^-28^ |
| UOO13421.1 |  | -3.15936 | 7.40E^-37^ |
| UOO15966.1 | *hlgA* | -2.96025 | 1.77E^-11^ |
| UOO13420.1 | *norB* | -2.74543 | 1.41E^-34^ |
| UOO15633.1 | *ecb* | -2.64187 | 9.96E^-20^ |
| UOO14551.1 | *argF* | -2.50964 | 1.08E^-08^ |
| UOO13584.1 | *pxpA* | -2.40651 | 3.57E^-12^ |
| UOO15648.1 |  | -2.40195 | 3.39E^-30^ |
| UOO13585.1 |  | -2.32447 | 2.13E^-18^ |
| UOO13586.1 |  | -2.29552 | 1.49E^-05^ |
| UOO14617.1 |  | -2.28675 | 2.25E^-05^ |
| UOO14552.1 | *arcA* | -2.25704 | 1.03E^-06^ |
| UOO14400.1 |  | -2.24601 | 4.62E^-05^ |
| UOO14401.1 |  | -2.23044 | 8.92E^-20^ |
| UOO14550.1 | *arcD* | -2.22392 | 4.16E^-10^ |
| UOO14341.1 | *hlgC* | -2.17782 | 3.40E^-08^ |
| UOO13587.1 |  | -2.15038 | 1.93E^-09^ |
| UOO14705.1 | *adhE* | -2.12793 | 2.43E^-13^ |
| UOO15798.1 |  | -2.04451 | 9.04E^-17^ |
| UOO13964.1 | *lukH* | -2.03271 | 3.02E^-09^ |
| UOO15157.1 |  | -1.99688 | 0.004857 |
| UOO13963.1 | *lukG* | -1.95638 | 7.79E^-07^ |
| UOO14813.1 | *lrgB* | -1.94945 | 1.40E^-14^ |
| UOO15637.1 | *scb* | -1.93314 | 2.10E^-11^ |
| UOO13583.1 |  | -1.92755 | 2.16E^-13^ |
| UOO14342.1 | *hlgB* | -1.92074 | 2.74E^-06^ |
| UOO14480.1 |  | -1.89148 | 2.55E^-15^ |
| UOO14662.1 |  | -1.81741 | 2.10E^-09^ |
| UOO14598.1 |  | -1.80603 | 0.010752 |
| UOO14615.1 |  | -1.80107 | 6.76E^-05^ |
| UOO14667.1 |  | -1.80094 | 1.63E^-08^ |
| UOO14549.1 | *arcC* | -1.79466 | 2.64E^-06^ |
| UOO14759.1 |  | -1.76974 | 2.03E^-07^ |
| UOO14812.1 | *lrgA* | -1.75502 | 1.31E^-07^ |
| UOO14548.1 |  | -1.69732 | 2.19E^-05^ |

**Table.S1** Differentially expressed genes of MH-grown and itaconate-adapted *S. aureus* ATCC29213.

| GID | name | log2(FC) | *p*.ajusted |
| --- | --- | --- | --- |
| UOO15349.1 |  | -1.69727 | 0.028804 |
| UOO15609.1 | *isdC* | -1.67846 | 0.01309 |
| UOO15636.1 | *efb* | -1.6642 | 2.77E^-05^ |
| UOO13588.1 | *pxpB* | -1.65797 | 9.95E^-06^ |
| UOO15799.1 |  | -1.61667 | 4.67E^-10^ |
| UOO14776.1 | *pflA* | -1.60174 | 2.02E^-10^ |
| UOO14616.1 |  | -1.58909 | 9.24E^-05^ |
| UOO15800.1 | *thrC* | -1.56635 | 2.72E^-09^ |
| UOO15812.1 |  | -1.54084 | 0.014286 |
| UOO15475.1 |  | -1.52568 | 0.021783 |
| UOO14458.1 |  | -1.51225 | 0.000104 |
| UOO15821.1 | *acnA* | -1.49368 | 2.46E^-27^ |
| UOO15209.1 |  | -1.49146 | 3.31E^-06^ |
| UOO14323.1 |  | -1.46271 | 1.03E^-14^ |
| UOO14514.1 |  | -1.45986 | 8.68E^-08^ |
| UOO13993.1 |  | -1.43477 | 0.001855 |
| UOO13994.1 | *leuB* | -1.41057 | 0.000779 |
| UOO14775.1 | *pflB* | -1.40868 | 5.70E^-08^ |
| UOO14758.1 |  | -1.40763 | 0.006503 |
| UOO13992.1 | *ilvC* | -1.39377 | 0.020887 |
| UOO14516.1 |  | -1.35479 | 0.001296 |
| UOO15367.1 |  | -1.34428 | 0.019009 |
| UOO15673.1 | *pyrR* | -1.33566 | 0.009391 |
| CDS |  | -1.32121 | 0.018666 |
| UOO15780.1 | *glnA* | -1.31849 | 0.000381 |
| UOO13907.1 | *sak* | -1.3068 | 1.72E^-08^ |
| UOO14896.1 |  | -1.29758 | 0.009269 |
| UOO15610.1 | *isdD* | -1.2966 | 0.015536 |
| UOO14350.1 |  | -1.28558 | 0.016753 |
| UOO14854.1 |  | -1.28084 | 0.000212 |
| UOO14501.1 |  | -1.27752 | 0.000104 |
| UOO15833.1 |  | -1.26068 | 0.017467 |
| UOO15781.1 |  | -1.23992 | 1.03E^-06^ |
| UOO15495.1 |  | -1.23357 | 0.007518 |
| UOO14771.1 | *uhpT* | -1.23296 | 0.000207 |
| UOO15908.1 |  | -1.22137 | 0.006971 |
| UOO14386.1 |  | -1.22023 | 0.006005 |
| UOO13995.1 | *leuC* | -1.20391 | 0.012128 |
| UOO15510.1 |  | -1.195 | 0.024504 |
| UOO14210.1 |  | -1.19067 | 1.08E^-08^ |

**Table.S1** Differentially expressed genes of MH-grown and itaconate-adapted *S. aureus* ATCC29213 (continue).

| GID | name | log2(FC) | *p*.ajusted |
| --- | --- | --- | --- |
| UOO14978.1 |  | -1.15907 | 0.005174 |
| UOO14979.1 |  | -1.15045 | 0.01572 |
| UOO13494.1 |  | -1.13842 | 0.00011 |
| UOO14895.1 |  | -1.13352 | 0.010525 |
| UOO14387.1 | *cntA* | -1.11993 | 8.25E^-05^ |
| UOO15220.1 |  | -1.11961 | 0.000173 |
| UOO14596.1 |  | -1.10442 | 0.002764 |
| UOO14912.1 |  | -1.09762 | 0.001486 |
| UOO14809.1 | *scdA* | -1.09577 | 2.74E^-06^ |
| UOO14891.1 |  | -1.09149 | 0.000282 |
| UOO15373.1 |  | -1.08417 | 0.006239 |
| UOO14981.1 | *aaa* | -1.07319 | 6.61E^-05^ |
| UOO13692.1 |  | -1.06974 | 0.016616 |
| UOO14498.1 |  | -1.06657 | 0.015315 |
| UOO14227.1 |  | -1.05664 | 0.00025 |
| UOO13997.1 | *ilvA* | -1.04 | 0.014094 |
| UOO14075.1 |  | -1.0322 | 0.004569 |
| UOO14721.1 | *isdI* | -1.03148 | 0.007684 |
| UOO14502.1 |  | -1.03077 | 0.000529 |
| UOO15674.1 |  | -1.01237 | 0.001579 |
| UOO15444.1 |  | -1.00845 | 0.012823 |
| UOO14631.1 | *hutH* | 1.009516 | 6.04E^-09^ |
| UOO14733.1 |  | 1.015232 | 1.22E^-09^ |
| UOO14740.1 |  | 1.031435 | 1.74E^-05^ |
| UOO14781.1 |  | 1.120993 | 0.027158 |
| UOO14782.1 |  | 1.11045 | 0.008712 |
| UOO14870.1 | *lip2* | 3.44975 | 5.10E^-46^ |
| UOO14879.1 |  | 1.68695 | 1.09E^-12^ |
| UOO14880.1 |  | 1.922266 | 8.99E^-14^ |
| UOO14881.1 |  | 2.036684 | 1.49E^-11^ |
| UOO14882.1 |  | 1.206496 | 7.62E^-08^ |
| UOO14969.1 |  | 1.330141 | 0.000236 |
| UOO15882.1 |  | 3.180671 | 4.66E^-19^ |
| UOO15883.1 |  | 3.220136 | 1.51E^-21^ |
| UOO15884.1 |  | 3.393307 | 4.91E^-23^ |
| UOO15155.1 |  | 1.11149 | 5.93E^-07^ |
| UOO15323.1 |  | 1.199174 | 1.41E^-10^ |
| UOO15446.1 |  | 1.814039 | 4.31E^-06^ |
| UOO15525.1 | *sspC* | 1.276208 | 0.003768 |
| UOO15527.1 | *sspA* | 1.181911 | 0.000742 |

**Table.S1** Differentially expressed genes of MH-grown and itaconate-adapted *S. aureus* ATCC29213 (continue).

| GID | name | log2(FC) | *p*.ajusted |
| --- | --- | --- | --- |
| UOO15575.1 | *lpdA* | 1.110445 | 1.08E^-08^ |
| UOO15640.1 | *hyl* | 1.343804 | 1.51E^-07^ |
| UOO15652.1 |  | 2.39184 | 1.81E^-09^ |
| UOO15653.1 |  | 2.063797 | 7.88E^-13^ |
| UOO13521.1 | *gcvPA* | 1.209032 | 5.90E^-12^ |
| UOO13522.1 | *gcvT* | 1.181688 | 3.03E^-06^ |
| UOO13651.1 | *rpmI* | 1.249279 | 0.030516 |
| UOO13853.1 | *ftnA* | 1.13684 | 8.67E-05 |
| UOO13905.1 | *scn* | 1.041712 | 0.006736 |
| UOO15946.1 | *hld* | 1.714424 | 8.99E^-14^ |
| UOO13976.1 |  | 1.043858 | 3.22E-06 |
| UOO15950.1 | *kdpB* | 1.0331 | 0.003198 |
| UOO14012.1 | *kdpA* | 1.285828 | 0.022324 |
| UOO14031.1 |  | 1.500393 | 7.25E^-12^ |
| UOO14109.1 | *sfaA* | 1.233875 | 6.23E-05 |
| UOO14110.1 | *sfaD* | 1.040273 | 0.000549 |
| UOO14215.1 | *ureC* | 1.295451 | 6.30E^-06^ |
| UOO14220.1 | *sarR* | 1.029054 | 4.50E-06 |
| UOO14284.1 |  | 3.264544 | 5.50E^-13^ |
| UOO14285.1 |  | 3.305224 | 1.04E^-22^ |
| UOO14346.1 | *bioA* | 2.300595 | 0.007106 |
| UOO14421.1 |  | 1.453811 | 4.04E^-11^ |
| UOO14422.1 | *gntK* | 1.237105 | 2.74E^-06^ |
| UOO14461.1 |  | 1.207231 | 0.009052 |
| UOO14570.1 | *sasA* | 1.396956 | 0.000148 |
| UOO14585.1 | *lip1* | 1.760449 | 1.61E^-07^ |

**Table. S2** The top 15 upregulated and downregulated metabolites.

| Metabolite | log2(FC) | *p*.ajusted |
| --- | --- | --- |
| Tetrac | -6.5165 | 0.035683 |
| [(2R)-1-icosanoyloxy-3-phosphonooxypropan-2-yl] 22-methyltetracosanoate | -6.1803 | 0.00094493 |
| gamma-L-Glutamyl-L-pipecolic acid | -5.9594 | 2.32E^-07^ |
| 2-Hydroxyethanesulfonic acid;6-methyl-7-nitro-5-(piperidin-1-ylmethyl)-1,4-dihydroquinoxaline-2,3-dione | -5.8599 | 0.021598 |
| PE(22:0/22:4(7Z,10Z,13Z,16Z)) | -5.6539 | 0.034275 |
| Dioleoylphosphatidylcholine | -5.5163 | 0.0032128 |
| Sitafloxacin hydrate | -5.2557 | 0.0010193 |
| Arginine | -5.1993 | 4.09E^-07^ |
| [(2R,3S,4S,5R,6S)-3,4,5-trihydroxy-6-[(6Z)-1-hydroxy-6-(2-hydroxy-2-methylpropylidene)-2,2,4,4-tetramethyl-3,5-dioxocyclohexyl]oxyoxan-2-yl]methyl 3,4,5-trihydroxybenzoate | -5.1467 | 0.00069315 |
| Acetylpterosin C | -5.0288 | 0.0001461 |
| Linagliptin Metabolite CD1790 | -4.9253 | 0.00026489 |
| 4-(4-Chlorophenyl)piperazine-1-carboximidamide | -4.6351 | 6.68E^-10^ |
| 4-tert-Butyl-N-(4-phenyl-thiazol-2-yl)-benzamide | -4.6342 | 0.0038095 |
| Epigallocatechin 3-O-cinnamate | -4.573 | 0.000015908 |
| PS(20:2(11Z,14Z)/22:4(7Z,10Z,13Z,16Z)) | -4.3361 | 0.000093813 |
| Artemoin C | 10.517 | 0.000189 |
| Brevetoxin A | 9.8911 | 0.000129 |
| 1-Pyrenesulfonic acid | 8.7674 | 0.002773 |
| Artemoin B | 8.4403 | 2.78E^-06^ |
| Mesoaconic acid | 8.2833 | 0.000876 |
| Pretyrosine | 8.2317 | 2.88E^-07^ |
| (-)-Neplanocin A | 7.4352 | 0.00019 |
| m-Cresol | 7.2901 | 3.49E-08 |
| Ceftin | 6.8694 | 1.03E^-05^ |
| Artemoin A | 6.7718 | 3.44E^-05^ |
| Tyr-Phe4Cl-OH | 6.7342 | 4.10E^-06^ |
| 10-(3-Piperazin-1-ylpropyl)-2-(trifluoromethyl)phenothiazine | 6.695 | 1.35E^-07^ |

**Table. S2** The top 15 upregulated and downregulated metabolites(continue) .

| Metabolite | log2(FC) | *p*.ajusted |
| --- | --- | --- |
| Porphobilinogen(1-) | 6.5109 | 6.12E^-07^ |
| 1,2,3-Trihydroxybenzene | 6.4019 | 1.74E^-08^ |
| Diethyl 2-methyl-3-oxosuccinate | 6.3898 | 1.93E^-06^ |

**Table.S3** **Primer used for RT-qPCR**

| **Primers** | **Sequences (5’-3’)** |
| --- | --- |
| *Irg1*-F | TGGGGCCTTTTATGCCAACT |
| *Irg1*-R | CTCACCTGTGGCCTGTTGAT |
| *Gapdh-F* | AGAACATCATCCCTGCCTCTACT |
| *Gapdh-R* | TGGACGGTTTATACTACTGTAG |


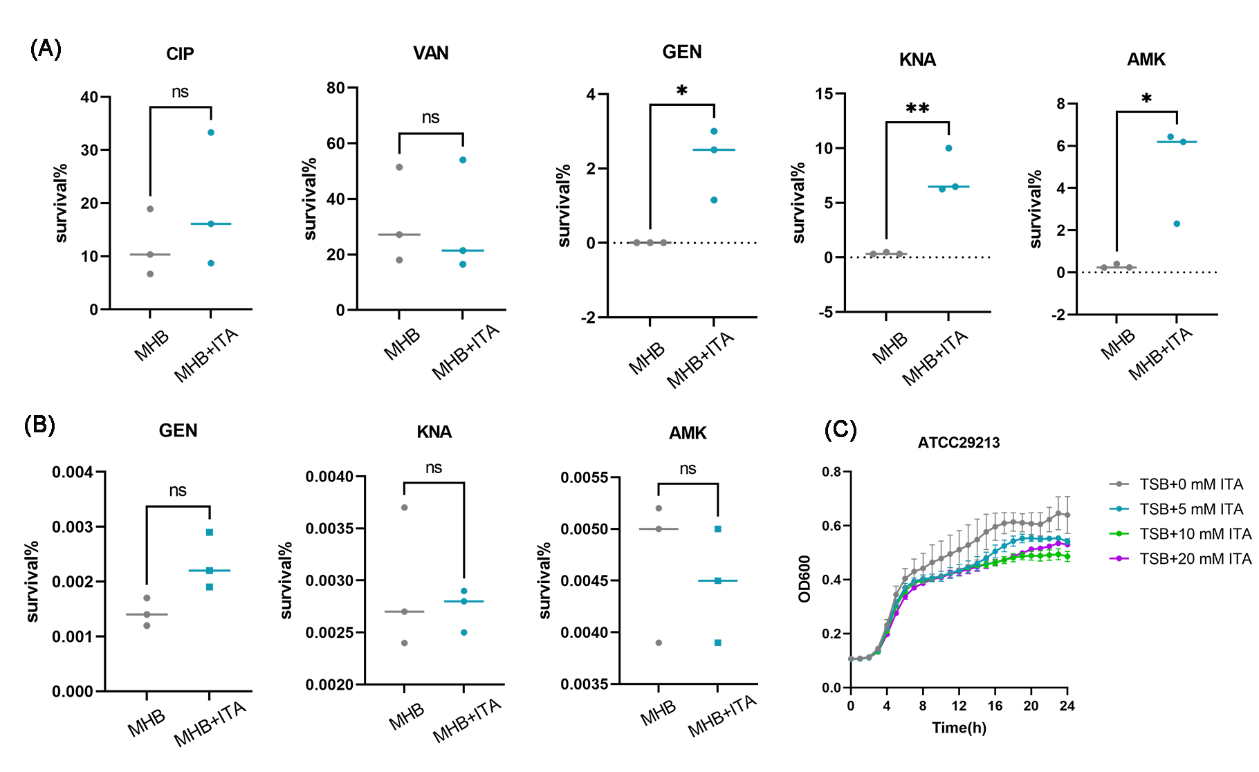


**Fig.S1:** **(A)** The survival rate of ATCC29213 after being challenged for 6 h with 10 μg/mL antibiotic (ciprofloxacin (CIP), vancomycin (VAN), gentamycin (GEN), kanamycin (KNA) amikacin (AMK)). **(B)** The survival rate of ATCC29213 after being challenged for 24 h with 10 μg/mL antibiotic (gentamycin (GEN), kanamycin (KNA) amikacin (AMK)). **(C)** Representative growth curves of ATCC29213 in TSB supplemented with itaconate(0-20mM) Data represent mean ± SD. Statistical significance was determined by unpaired t-test (two-tailed) or one-way ANOVA with Sidak’s multiple comparison. *p<0.05 **p<0.01.


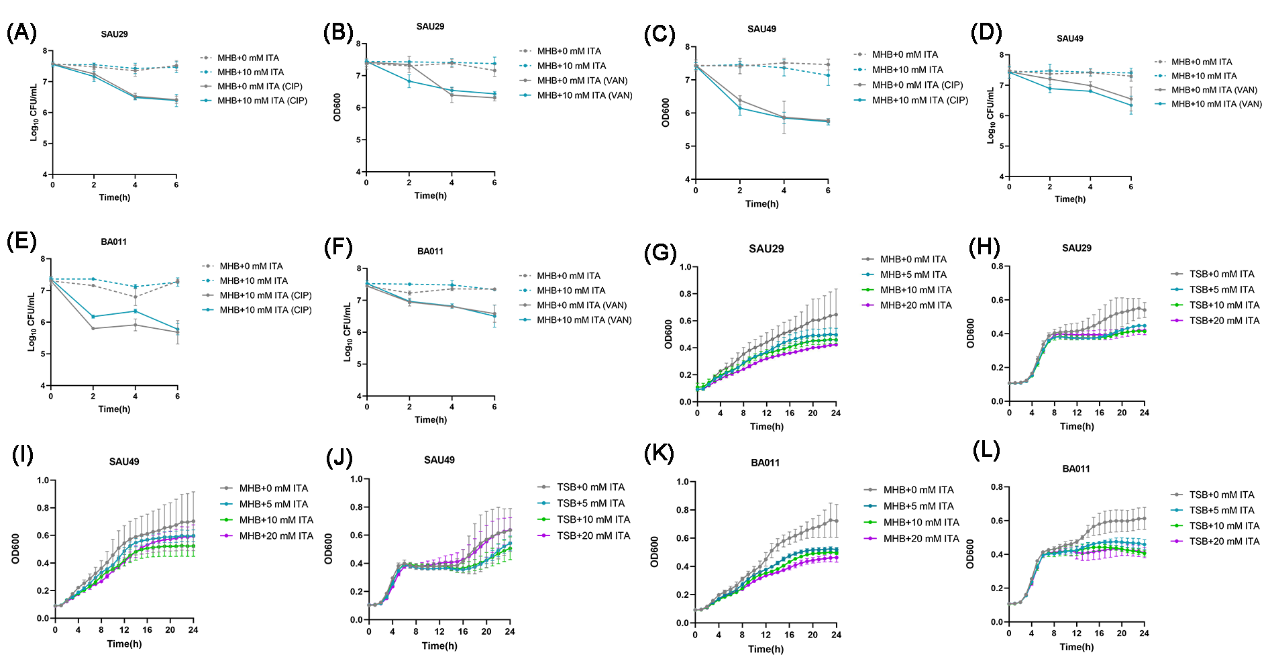


**Fig.S2: (A-B)** The time-dependent killing pattern of SAU29 in the MHB-grown group and the itaconate-adapted group over 6 h. The graphs show log10 CFU /ml of *S. aureus* challenged with 10 μg/mL antibiotic (ciprofloxacin (CIP), vancomycin (VAN),). **(C-D)** The time-dependent killing pattern of SAU49 in the MHB-grown group and the itaconate-adapted group over 6 h. The graphs show log10 CFU /ml of *S. aureus* challenged with 10 μg/mL antibiotic (ciprofloxacin (CIP), vancomycin (VAN),). **(E-F)** The time-dependent killing pattern of BA011 in the MHB-grown group and the itaconate-adapted group over 6 h. The graphs show log10 CFU /ml of *S. aureus* challenged with 10 μg/mL antibiotic (ciprofloxacin (CIP), vancomycin (VAN),). (**G-L**) Representative growth curves of SAU29, SAU49, BA011 in MHB (or TSB) supplemented with itaconate (0-20mM)


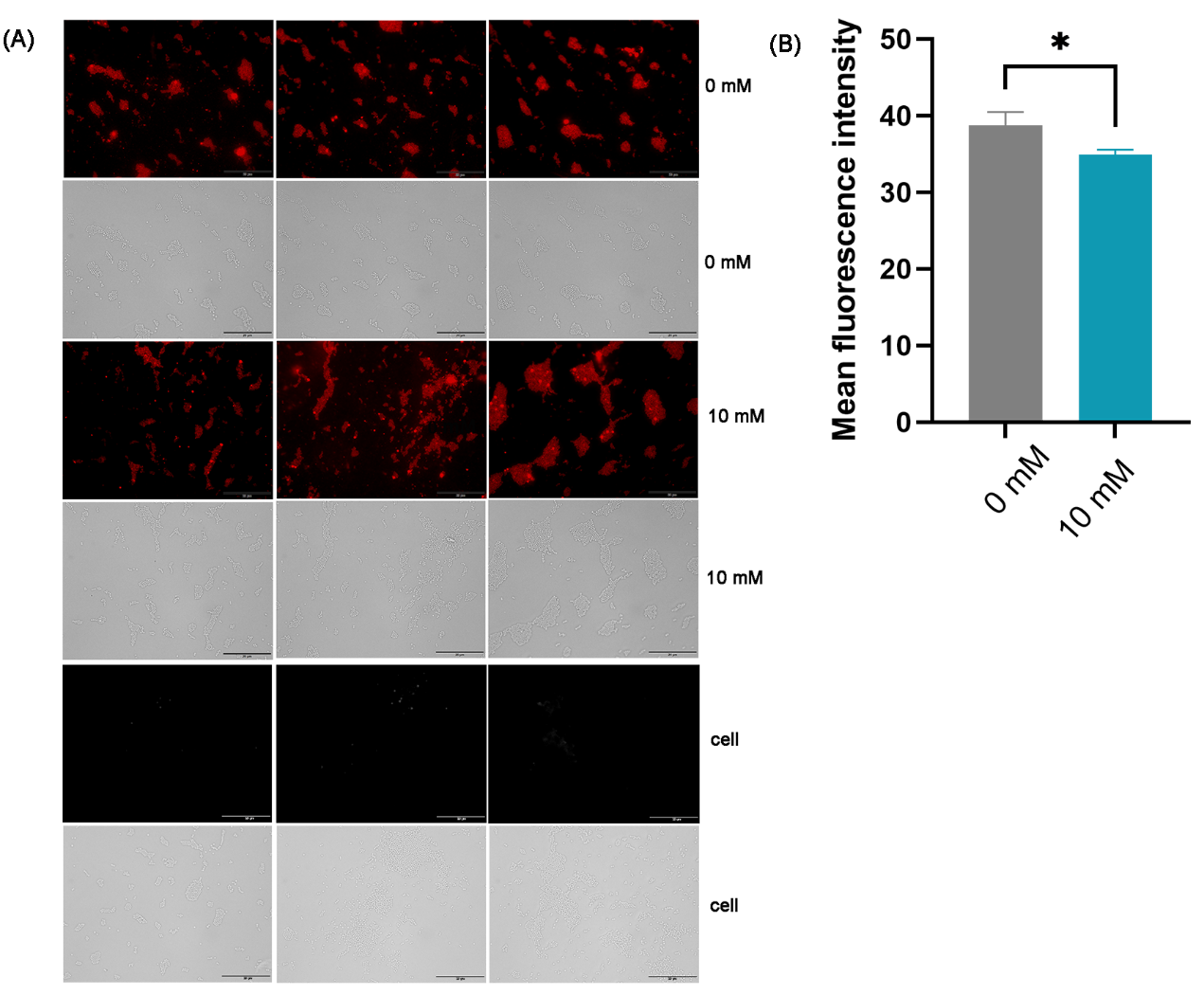


**Fig.S3:** (**A**) The images of the itaconate group and the MHB-grown group cells. (**B**) The result showed that the mean fluorescence intensity of the images in the MHB-grown group and the itaconate-adapted group.


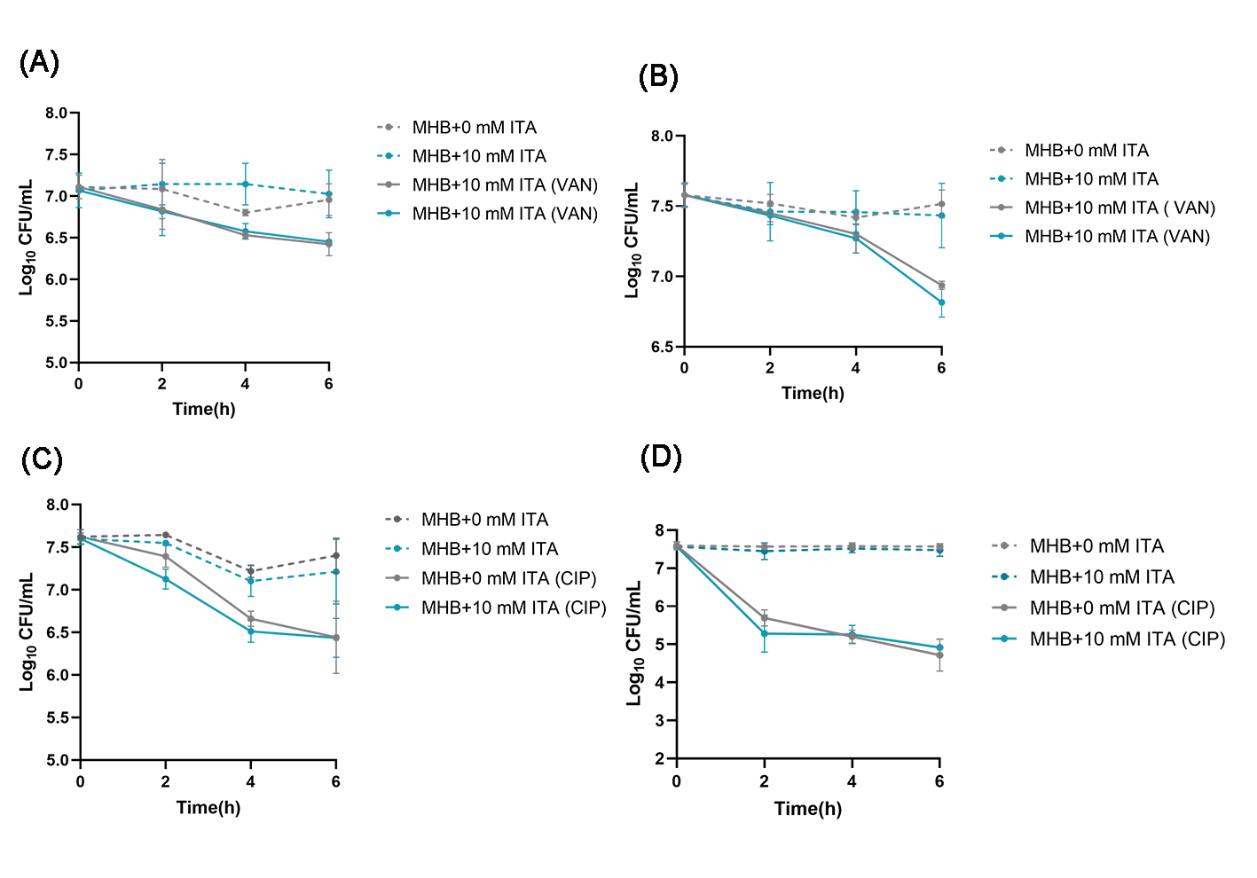


**Fig.S4:** The time-dependent killing pattern of S. aureus ATCC29213 in the MHB-grown group and the itaconate-adapted group over 6 h. The graphs show log10 CFU /ml of S. aureus challenged with 10 μg/mL vancomycin (**A**), 40 μg/mL vancomycin (**B**), 10 μg/mL ciprofloxacin (**C**) and 2.5 μg/mL ciprofloxacin (**D**).
